# Tactile Stimulation Evokes Long-Lasting Potentiation of Purkinje Cell Discharge *In Vivo*

**DOI:** 10.3389/fncel.2016.00036

**Published:** 2016-02-18

**Authors:** K. B. Ramakrishnan, Kai Voges, Licia De Propris, Chris I. De Zeeuw, Egidio D’Angelo

**Affiliations:** ^1^Department of Brain and Behavioral Sciences, University of PaviaPavia, Italy; ^2^Consorzio Interuniversitario per le Scienze Fisiche della Materia (CNISM)Pavia, Italy; ^3^Department of Neuroscience, Erasmus University RotterdamRotterdam, Netherlands; ^4^Netherlands Institute for Neuroscience, Royal Academy of Arts and SciencesAmsterdam, Netherlands; ^5^Brain Connectivity Center, Istituto Neurologico IRCCS Fondazione C. MondinoPavia, Italy

**Keywords:** Purkinje cell, molecular layer interneurons, LTP, LTD, suppression, cerebellum, *in vivo* electrophysiology

## Abstract

In the cerebellar network, a precise relationship between plasticity and neuronal discharge has been predicted. However, the potential generation of persistent changes in Purkinje cell (PC) spike discharge as a consequence of plasticity following natural stimulation patterns has not been clearly determined. Here, we show that facial tactile stimuli organized in theta-patterns can induce stereotyped N-methyl-D-aspartate (NMDA) and gamma-aminobutyric acid (GABA-A) receptor-dependent changes in PCs and molecular layer interneurons (MLIs) firing: invariably, all PCs showed a long-lasting increase (*Spike-Related Potentiation* or SR-P) and MLIs a long-lasting decrease (*Spike-Related Suppression* or SR-S) in baseline activity and spike response probability. These observations suggests that tactile sensory stimulation engages multiple long-term plastic changes that are distributed along the mossy fiber-parallel fiber (MF-PF) pathway and operate synergistically to potentiate spike generation in PCs. In contrast, theta-pattern electrical stimulation (ES) of PFs indistinctly induced SR-P and SR-S both in PCs and MLIs, suggesting that tactile sensory stimulation preordinates plasticity upstream of the PF-PC synapse. All these effects occurred in the absence of complex spike changes, supporting the theoretical prediction that PC activity is potentiated when the MF-PF system is activated in the absence of conjunctive climbing fiber (CF) activity.

## Introduction

Long-term modifications in synaptic transmission (either long-term potentiation or depression, LTP or LTD) and in neuronal intrinsic excitability, collectively called plasticity, are thought to provide a critical mechanism for learning and memory in brain circuits (Bliss and Collingridge, 1993). The main consequence of plasticity is to regulate neuronal spike discharge in response to specific inputs and therefore to modify neural circuit operations. In the cerebellar network, a precise relationship between plasticity and neuronal discharge has been predicted. According to the “motor learning theory”, plasticity at the parallel fiber (PF)—Purkinje cell (PC) synapse is essential to regulate the PC output and motor learning (Marr, 1969; Albus, 1971). Whereas, LTD should be driven by error-related signals carried by climbing fibers (CFs; Ito and Kano, 1982), LTP should occur when such error signals are absent (Sakurai, 1987). This double mechanism of learning is thought to provide the key for understanding cerebellar functioning (Doya, 1999). However, quite surprisingly, the generation of persistent changes in PC spike discharge as a consequence of plasticity following natural neuronal activity patterns has not been clearly determined.

Following the initial description of classical PF-PC LTP and LTD, numerous other forms of synaptic and non-synaptic plasticity have been reported *in vitro* in the granular layer, molecular layer and cerebellar nuclei (Hansel et al., 2001; Gao et al., 2012; D’Angelo, 2014). Various types of PF-PC LTD have been observed using different stimulus paradigms and baseline settings (Schonewille et al., 2011), so that classical LTD could be just one form of flexible bidirectional mechanism that in fact depends on the precise pattern, phase and intensity of PF activity (Coesmans et al., 2004). Likewise, PF-PC LTP, which is caused by PF activity alone, could well be expressed both pre- and postsynaptically and to some extent be converted into LTD when PF activity patterns are changed (Salin et al., 1996; Lev-Ram et al., 2003; Qiu and Knöpfel, 2007). The same PF activity patterns can also influence plasticity of intrinsic excitability in PCs (Belmeguenai et al., 2010). Bidirectional synaptic plasticity changes, as well as changes in intrinsic excitability, also occur in granule cells (D’Errico et al., 2009) and in the MLI circuit (Rancillac and Crépel, 2004; Tanaka et al., 2013). Thus, the spike firing changes that might ultimately occur in PCs *in vivo* following specific stimulus patterns remain hard to predict and may be determined by the convergence of multiple elementary forms of plasticity distributed across the entire afferent mossy fiber-parallel fiber (MF-PF) pathway.

*In vivo*, persistent depression of PF-PC transmission (Ito and Kano, 1982; Gao et al., 2003; Wang et al., 2009; Márquez-Ruiz and Cheron, 2012) and of intrinsic changes in PC excitability (Johansson et al., 2014) have been supported by a variety of experimental approaches all using electrical stimulation (ES) of PFs and CFs. Moreover, reciprocal bidirectional plasticity of PF receptive fields has been reported in PCs and their afferent molecular layer interneurons (MILs) following sensory stimulation (Jörntell and Ekerot, 2002) as well as in the granular layer (Roggeri et al., 2008). However, to date no evidence is available for long-term changes in PC spike discharge in response to natural stimulation patterns. Here we show, in rats, that entrainments of facial tactile stimulation (TS) can induce stable potentiation (referred to as *Spike-Related Potentiation* or SR-P) of PC firing combined with stable suppression (*Spike-Related Suppression* or SR-S) of MLI firing. These observations, complemented by the analysis of N-methyl-D-aspartate (NMDA) and gamma-aminobutyric acid (GABA-A) receptor blockage, highlight the concept that cerebellar plasticity is distributed along the MF-PF pathway and operates synergistically to potentiate PC firing.

## Materials and Methods

Multiple single-unit recordings were performed from the cerebellar cortex in 24–30 days-old Wistar rats under urethane anesthesia (Roggeri et al., 2008). Urethane (Sigma-Aldrich) has been reported to exert its anesthetic action through multiple weak effects, including a 10% reduction of NMDA, an 18% reduction of AMPA and a 23% enhancement of GABA-A receptor-mediated currents (Hara and Harris, 2002). Urethane was preferred to ketamine or isoflurane, because these latter drugs largely exert their action by blocking NMDA receptors (up to 80 and 60%, respectively; Hara and Harris, 2002) and therefore potentially favor induction of long-term depression (Godaux et al., 1990; Márquez-Ruiz and Cheron, 2012).

### Surgical Procedures

Animals were deeply anesthetized with intraperitoneal injections of urethane. Induction (1.35 g/kg urethane dissolved in 0.9% NaCl) was followed 30 min later by a booster injection (10% of the induction dose) in order to stabilize anesthesia. The level of anesthesia was constantly monitored by evaluating spontaneous whisking and the intensity of leg withdrawal after pinching. The animal was placed on a stereotaxic table (David Kopf Instuments, Tujunga, CA, USA) covered with a heating pad and body temperature was monitored with a rectal probe and maintained at 37 ± 0.5°C through a feedback temperature controller (Fine Scientific Tools Inc., Foster City, CA, USA). Lidocaine (0.2 ml; Astrazeneca) was applied subcutaneously above the right cerebellum (−6.36 mm AP and −2.56 mm ML to bregma), the skin and muscles were removed, and a subsequent craniotomy of the occipital bone exposed the surface of Crus-I and Crus-II (Bower and Woolston, 1983). The *dura mater* was carefully removed and the cerebellar surface was covered with standard extracellular Kreb’s solution [in mM: NaCl (120), KCl (2), MgSO_4_ (1.2), NaHCO_3_ (26), KH_2_PO_4_ (1.18), CaCl_2_ (2) and glucose (11)] pre-warmed at 37°C, carboxygenated and equilibrated at a pH of 7.4.

### *In Vivo* Electrophysiology and Pharmacology

Quartz-coated platinum-tungsten fiber electrodes (2–5 MΩ; Thomas Recording, Giessen, Germany) were used for neuronal recordings. Recording electrodes were lowered into the PC layer from the cortical surface in the Crus-I or Crus-II area, which are known to receive the somatosensory inputs coming primarily from the facial and whisker pad areas (Bosman et al., 2010). The electrophysiological signals were digitized at 25 kHz, using a 20–6000 Hz band-pass filter, amplified and stored using a RZ5D processor multi-channel workstation (Tucker-Davis Technologies, Alachua, FL, USA). Single-unit recordings were collected both as spontaneous signals and stimulus-evoked responses in spike trains.

All drugs were prepared in extracellular Kreb’s solution and superfused in a subset of experiments onto the cerebellar surface [100 μM gabazine (SR95531) and 100 μM APV; Sigma-Aldrich]. The drugs were applied immediately after the control recording period and then maintained throughout.

### Sensory and Electrical Stimulation

Tactile sensory stimulation was performed using air-puffs (30 ms pulses delivered at 30–60 psi; Chadderton et al., 2004; Roggeri et al., 2008) delivered through a small tube ending with a nozzle of 0.5 mm diameter connected to a MPPI-2 pressure injector (Applied Scientific Instrumentation, Eugene, OR, USA) positioned 2–3 mm away from the snout area of the animal. Once the extracellular signals were detected, the nozzle was moved over the peri-oral region to activate the corresponding receptive field and to optimize and stabilize the neuronal response. Following a 20 min monitoring period (0.5 Hz), a theta sensory stimulation (TSS) entrainment pattern (a burst of 80 air puffs delivered at 4 Hz) was delivered to induce plasticity. Then, 0.5 Hz monitoring was continued for at least 40 min. Recordings lasting for less than 40 min after induction were not considered for further analyses.

ES (100 μs, 10–15 V pulses) was performed using a co-axial platinum bi-polar electrode (Micro Probe) connected to an analog pulse generator through an isolation unit. The electrode was placed into the molecular layer 100–150 μm below the cortical surface in order to stimulate a PF bundle. The monitoring pattern consisted of repeated electrical stimuli delivered at 0.5 Hz. Following a 20 min control period, a theta burst stimulation (TBS) pattern (made of 4 Hz bursts made of 1, 2 or 3 pulses at 100 Hz) was delivered to induce plasticity. Then, monitoring was continued for at least 40 min. Recordings lasting for less than 40 min after induction were not considered for analysis.

### Data Analyses

Recordings were pre-analyzed online using Openex (Tucker-Davis Technologies) and then re-analyzed offline using custom-written software in MATLAB and Excel. Openscope (from Openex suite) was used to construct in real-time a peri-stimulus time histogram (PSTH) triggered by the air-puffs and to identify the stimulus-elicited responses online. The recorded spike trains were sorted out offline using SpikeTrain (Neurasmus BV, Rotterdam, Netherlands), running under MATLAB (Mathworks, MA, USA). PCs were identified by their spiking nature, the depth of the recording site from the cortical surface (ranging from 150–350 μm) and the coexistence of simple spikes (SSs) and complex spikes (CSs). CSs were recognized based on the presence of spikelets following the initial spike and a subsequent pause in SS firing. MLIs were identified by the absence of CSs, and normally showed more irregular and slower spike firing than PCs and were found at a slightly minor depth (100–250 μm; for details of identification, see Ruigrok et al., 2011; Badura et al., 2013). The stability of recordings was carefully considered and only the recordings in which the unit spike size was stable for at least one hour (±20%) were considered for further analyses. PSTH and raster plots were constructed both for PC SSs and CSs as well as MLI spikes using short bin widths (1, 2 or 5 ms) to allow the identification of the early peak of activity typical of the trigemino-cerebellar pathway (Bower and Woolston, 1983). The PSTH peak amplitude was measured relative to the preceding 500 ms baseline, the PSTH peak delay was measured relative to the time of stimulus onset, and the duration of the PSTH peak was measured at half-width. The baseline was used to measure the basal firing of the PC and MLI units. Statistical comparisons were carried out using the Student’s *t*-test (two-tailed or when allowed one-tailed) or one-way and multiple ANOVA. Data in the text are reported as mean ± SEM.

## Results

### PC and MLI Units Show Short-Latency Baseline Responses to Tactile Stimulation

**Figure 1:**
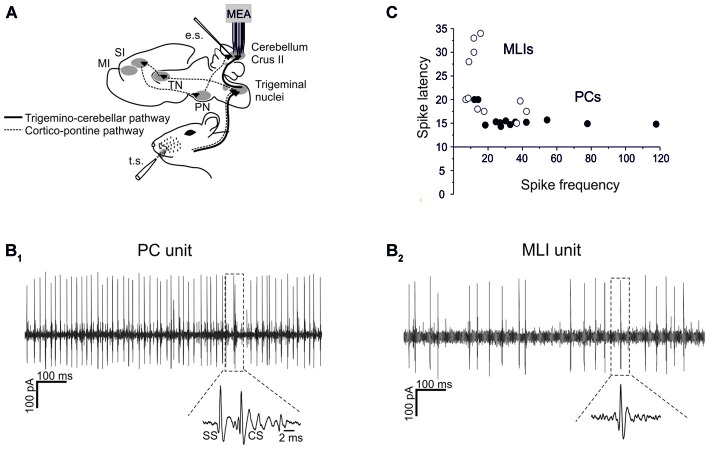
**Single-unit recordings from PCs and MLIs. (A)** Schematic representation of recording set-up. PC and MLI units were recorded from cerebellar Crus I and II in urethane anesthetized rats using a 16-channel multi-electrode array. Tactile stimulation (TS) was performed by delivering air puffs to the whisker pad and peri-oral area. Electrical stimulation (ES) of PFs was performed with a bi-polar tungsten electrode. The pathways involved in the transmission of sensory stimulation to the cerebellar cortex are shown, including the faster trigemino-cerebellar pathway and the slower thalamo-cortico-pontine pathway. **(B_1_,B_2_)** Sample spike trains recorded from a PC and an MLI are shown. In **(B_1_)** a PC unit shows relatively fast and regular SS firing with a CS followed by a SS pause (zoomed in the inset). In **(B_2_)** an MLI unit shows relatively less regular and slower firing compared to SS firing in the PC. **(C)** Spike delays (taken from PSTH as in Figure 2) are plotted against basal firing frequency. PCs usually show a higher firing frequency and a shorter spike latency than MLIs.

Single-unit recordings of PCs and MLIs were performed from Crus-I and Crus-II of the cerebellar cortex in anesthetized P24–30 rats (Figures 1A,B). All these units were spontaneously active, with PCs normally showing higher basal frequency than MLIs (47.8 ± 5.4 Hz, *n* = 13 vs. 18.7 ± 2.4 Hz, *n* = 11; *p* < 0.05, unpaired Student’s *t*-test; Figure 1C).

**Figure 2:**
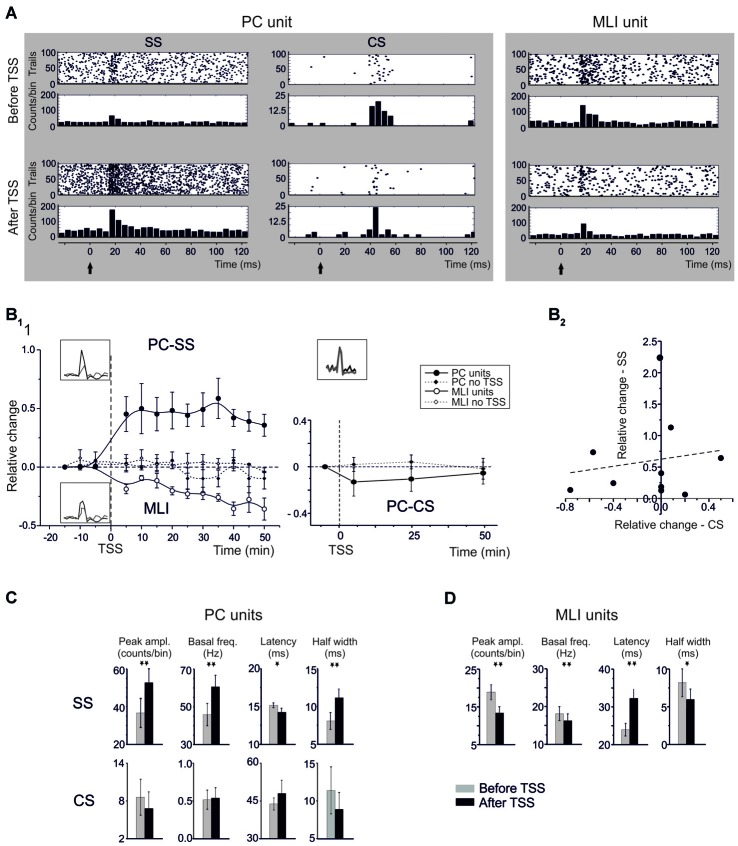
**Spike-related plasticity in PCs and MLIs following sensory stimulation. (A)** Raster plot and PSTH of a representative PC (left panel) and MLI (right panel) responding to air-puff stimulation. The raster plot and PSTH is made from 100 consecutive sweeps recorded before (upper panels) and after (bottom panels) TSS application. The PCs response is reported for SS and CS separately. TSS increases the number of SS generating a dense cloud immediately after the stimulus onset (black arrow) in the raster plot and increasing the PSTH peak (5 ms time bin). Conversely, TSS does not significantly influence the CS response. The MLI response shows a significant decrease in the number of spikes generating a sparse cloud immediately after the stimulus onset (black arrow) in the raster plot and decreasing the PSTH peak. **(B)** Time course of the normalized changes of PSTH peak amplitude (mean ± MSE). In the left plot, SS spike firing in PCs (*n* = 13) and MLI (*n* = 11) firing are compared to control recordings in which TSS was not applied (PC: *n* = 6; MLI: *n* = 4). After TSS, PCs units show SR-P, while MLI units show SR-S*.* In the right plot, CS firing changes after TSS (*n* = 9) are compared to control experiments in which TSS was not applied (*n* = 6). No significant changes occurred in the CS response. **(C)** Relative change of SSs firing frequency after TSS plotted against the relative changes of CSs firing frequency after TSS. The plot demonstrates absence of correlation between SS and CS changes after TSS. The dotted line is a linear regression on the data-set (adj. *R^2^* = −0.096, *prob* (>F) = 0.66). **(D)** The histograms show variations (mean ± MSE) of peak amplitude, latency and duration of the PSTH peak as well as of basal frequency for both PC SS and CS and for MLI. *p < 0.05, **p < 0.01, otherwise NS.

Both types of cells showed robust responses to whisker pad and snout area stimulation with short air-puffs (Figure 2). The PSTH of PCs typically showed an early SS response (15.6 ± 0.4 ms, *n* = 13), surpassing background activity and generating the PSTH peak. Then the PSTH slowly decayed toward basal frequency levels in about 30 ms (Figure 2A). This latency and decay of the SS PSTH were well in line with the delays of the signals known to be transmitted through the trigemino-cerebellar pathway and from granule cells to PCs (Bower and Woolston, 1983; Jaeger and Bower, 1994; Roggeri et al., 2008). CS responses generally occurred slightly later (40–50 ms; average 44.6 ± 3.3 ms; Figure 2A). Similarly to the PC SS responses, the MLIs also showed a monophasic PSTH peak (Figure 2A). The average latency of MLI responses was 23.1 ± 2.1 ms (*n* = 11), which was slightly, but significantly longer than that of the SS responses (unpaired *t*-test, *p* < 0.005).

### Theta Sensory Stimulation Induces Opposite Plasticity in PC Simple Spikes and MLI Units

TSS *in vivo* was previously shown to induce long-term changes in evoked local field potentials (LFPs) in the cerebellar granular layer (Roggeri et al., 2008). To investigate to what extent the impact of TSS propagated into the molecular layer, TSS was applied during molecular layer recordings and the corresponding spike-related changes were evaluated from PC and MLI PSTHs (Figure 2). Figure 2A shows raster plots and PSTHs, Figure 2B_1_ the time course of changes, and Figure 2C the average values of PC and MLI spike parameters taken in the 20–30 min period after TSS. Following TSS, all PC units (13/13) showed a significant increase in the probability of SS firing, or *Spike-Related Potentiation (SR-P)*, in that the peak amplitude of the SS PSTH increased by 46.8 ± 9.1% (*n* = 13; *p* < 0.0013, paired Student’s *t*-test; Figure 2A). At the same time, PSTH peak latency decreased by 9.0 ± 3.9% (*n* = 13; *p* < 0.039, paired Student’s *t*-test) and half-width increased by 32.3 ± 11.6% (*n* = 13; *p* < 0.012, paired Student’s *t*-test). Moreover, in the same cells, there was a persistent 37.8 ± 10.8% increase in basal SS frequency (*n* = 13; *p* < 0.002, paired Student’s *t*-test).

Noticeably, all MLI units (11/11) also showed a significant change in firing probability following TSS, but here we observed consistently a decrease, or *Spike-Related Suppression (SR-S)*, rather than an increase in spike discharge. The PSTH peak amplitude of the MLI spikes significantly decreased by 31.0 ± 4.8% (*n* = 11; *p* < 0.002, paired Student’s *t*-test). At the same time, PSTH peak latency increased by 34.6 ± 10.2% (*n* = 11; *p* < 0.01, paired Student’s *t*-test) and PSTH half-width decreased by 13.0 ± 5.5% (*n* = 11; *p* < 0.042, paired Student’s *t*-test). In addition, in the same cells, there was a 10.5 ± 3.5% decrease in basal spike frequency (*n* = 11; *p* < 0.014, one-tailed Student’s *t-test*).

The changes in firing probability of PCs and MLIs following TSS both persisted until the end of recordings (Figure 2B_1_). In contrast to the consistent modifications in spike-related parameters observed following TSS, no remarkable changes occurred in control groups of six PCs and four MLIs that were recorded for the same duration but without TSS (no statistical significance in any of the measured parameters; see Figure 2B).

### TSS does not Influence the CS Evoked Response

Whereas, many parameters of the SS responses changed dramatically following TSS, those of the CS responses did not (Figure 2). The change in relative PSTH peak amplitude of the CS responses (−3.0 ± 12.5% after TSS, *n* = 10; *p* = 0.57, paired Student’s *t*-test) as well as that of their latency of the PSTH peak amplitude (9.4 ± 10.1% after TSS, *n* = 10; *p* = 0.57, paired Student’s *t*-test) and PSTH duration at half-width (11.67 ± 8.7 after TSS, *n* = 10; *p* = 0.28, paired Student’s *t*-test) could hardly be detected. In addition, the basal CS firing frequency did not change significantly after TSS (6.1 ± 5.0%, *n* = 10; *p* = 0.49, paired Student’s *t*-test). Together these data indicate that, in contrast to the SS responses, the CS responses did not change substantially (for a direct comparison, also see Figure 2C). The slight non-significant decrease in CF frequency visible in Figure 2 might represent a temporary adaptation of IO discharge following repetitive stimulation.

The firing probability of PC CSs did not differ from that in a control group of six PCs that were recorded for the same duration but without TSS (*n* = 6, *p* > 0.2 for any of the measured parameters; see Figure 2B_1_). It should also be noted that following TSS there was no correlation between changes in CS and SS probability on a trial-by-trial basis (Figure 2B_2_), suggesting that CS did not contribute directly to the SS changes.

### Electrical Theta-Burst Stimulation of Parallel Fibers Induces Similar Plasticity in PC Simple Spikes and MLI Units

**Figure 3:**
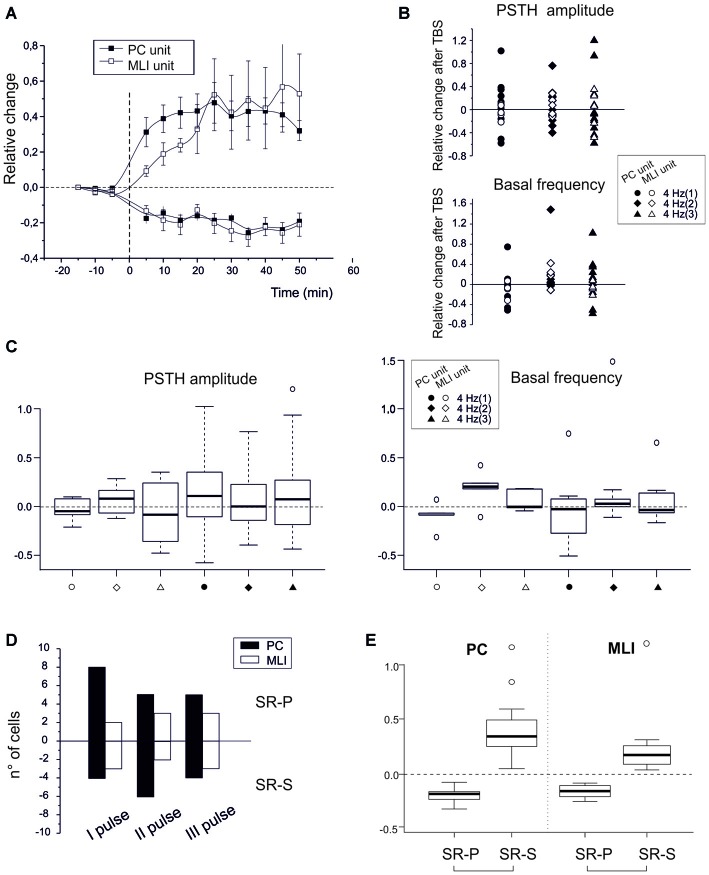
**Spike-related plasticity in PCs and MLIs following PF electrical stimulation. (A)** Time course of the normalized PSTH peak amplitude changes after PF TBS with singlets, doublets and triplets stimulation. Left, average time course of changes (mean ± MSE) in PCs (*n* = 32) and MLIs (*n* = 16) grouped depending on whether they made SR-P (18 PCs, 8 MLIs) or SR-S (14 PCs, 8 MLIs). Right, changes in individual units after TBS both for peak PSTH amplitude changes and for basal frequency changes. **(C)** Boxplot representation and one-way ANOVA on the samples reported in **(B)**. The Tuckey *post hoc* test did not reveal any significant differences between conditions. **(D)** Histogram representing the number of cells showing SR-S or SR-P with TBS protocols using singlets, doublets or triplets. **(E)** Boxplot representation and one-way ANOVA on the relative change of discharges reported in **(A)**. The Tuckey *post hoc* test revealed significant differences between SR-P and SR-S both for PCs and MLI (for numbers, see main text).

To isolate the molecular layer component of spike-related long-term plasticity in PC and MLI units, different theta-burst stimulation (TBS) patterns were electrically delivered to PFs (Figures 3A,B). These artificial TBS patterns consisted of single pulses, doublets or triplets at 100 Hz to imitate the most probable outputs of GCs in reaction to air-puff stimuli (Chadderton et al., 2004). The PCs (*n* = 32) responded with SSs to PF ES with a latency of 5.0 ± 0.4 ms (single pulses), 4.8 ± 0.3 ms (doublets) or 4.8 ± 0.4 ms (triplets). Independent from the number of pulses in TBS, the PC units showed SR-P (*n* = 18) or SR-S (*n* = 14) at comparable levels (SR-S in PCs −19.3 ± 3.4, *p* < 0.0001; SR-P in PCs 40.2 ± 8.3, *p* < 0.0001). Similarly, TBS induced SR-P (*n* = 8) or SR-S (*n* = 8) in the MLI units (*n* = 16) at similar amounts (SR-S in MLI −20.8 ± 5.2%, *p* = 0.0007; SR-P in MLI 38.3 ± 16.1%, *p* = 0.03). At the statistical level, there was no difference among any of the groups in multiple ANOVA tests (all *p*-values > 0.2; Figures 3C,D). In contrast, there were significant differences in multiple ANOVA tests when comparing the relative change of discharges of the PCs that showed SR-P responses with that of the PCs that showed SR-S responses (*p* < 0.001) as well as when comparing the same parameter between SR-P MLIs and SR-S MLIs (*p* < 0.0003; Figure 3E). Thus, just by switching from a natural entrainment paradigm engaging peripheral TS to an artificial paradigm employing ES of PFs in the molecular layer, the distribution of long-term plasticity changed from pure SR-P in PCs and pure SR-S in MLIs to a random occurrence of SR-P and SR-S in both PCs and MLIs.

### Synergistic Plasticity in PC Simple Spikes and MLI Units Induced by TSS can be Affected by Pharmacological Means

**Figure 4:**
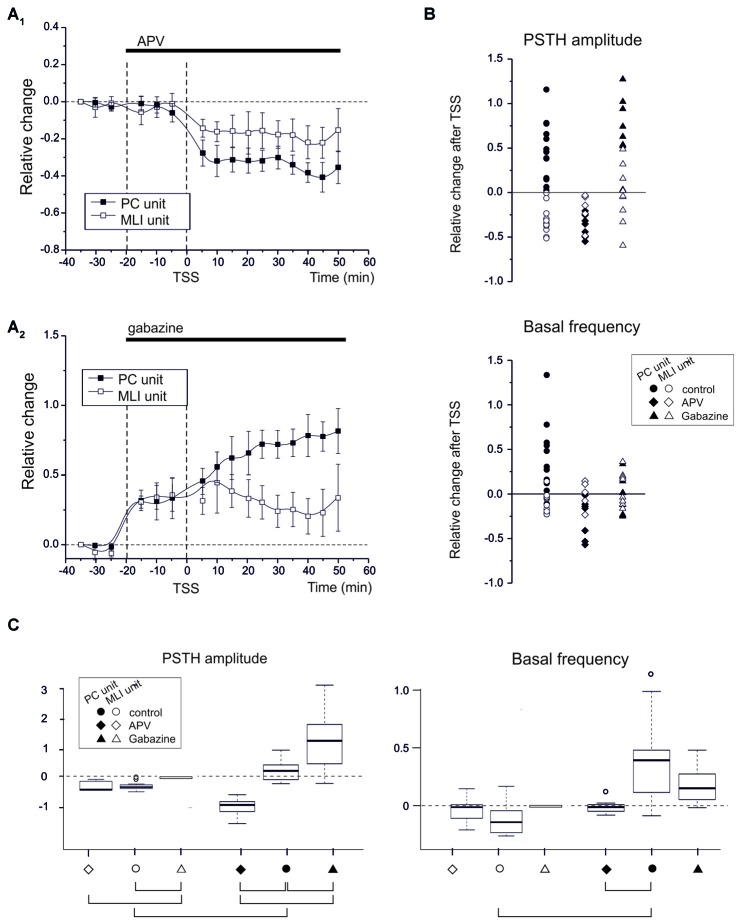
**NMDA and GABA receptor blockage differentially affect PC and MLI spike-related plasticity.** **(A_1_)** APV superfusion turns SR-P into SR-S in PCs but does not influence SR-S of MLIs. Average time course of changes (mean ± MSE) in PCs (*n* = 7) and MLIs (*n* = 5). **(A_2_)** Gabazine superfusion does not prevent SR-P in PCs but prevents SR-S in MLIs. Average time course of changes (mean ± MSE) in PCs (*n* = 8) and MLIs (*n* = 8). **(B)** Changes in individual units after TSS both for peak PSTH amplitude changes and for basal frequency changes. The different data sets compare control (as in Figure 2) to APV and gabazine (as in **A**). The values for APV and gabazine data sets are calculated with respect to the previous drug application period. **(C)** Boxplot representation and oneway ANOVA on the samples reported in **(B)**. The Tuckey *post hoc* test demonstrates significant differences between conditions (solid connectors). For *PSTH amplitude*: control PC vs. control MLI (*p* < 0.0002), control vs. APV in PC (*p* < 0.0007), control vs. gabazine in PC (*p* < 0.02). For *basal frequency*: control PC vs. control MLI (*p* < 0.01). Other diffenrences were not statistically significant (dashed connectors).

If the emerging hypothesis is correct, in that various forms of synaptic plasticity in the granular and molecular layer work together following natural entrainment, one should be able to affect this synergy by manipulating the activity of various types of synaptic receptors. Thus, since NMDA receptors play a key role in several forms of cerebellar synaptic plasticity, including those of afferents to GCs, PCs and MLIs (Qiu and Knöpfel, 2007; D’Errico et al., 2009; Piochon et al., 2010; D’Angelo, 2014; see also Carter and Regehr, 2000; Rossi et al., 2012), we repeated the TSS experiments in the presence of the competitive NMDA receptor antagonist, APV (100 μM), on the cerebellar surface. APV was continuously superfused before and after TSS (Figure 4A). During these experiments we identified seven PC units and five MLI units. Before TSS APV did not cause any significant change in PSTH peak amplitude (PCs −0.8 ± 1.2%; *n* = 7, *p* = 0.6; MLIs −1.8 ± 1.5%, *n* = 5, *p* = 0.8) or basal frequency (PCs −7.3 ± 1.4%; *n* = 7, *p* = 0.3; MLIs −18.8 ± 9.4%, *n* = 5, *p* = 0.6). However after TSS, APV application did alter the normal patterns of changes in that all the PC units showed SR-S in the PSTH peak (−34.8 ± 4.9%; *n* = 7, *p* < 0.000) and basal frequency (−27.7 ± 6.4%; *n* = 7, *p* = 0.004) and that the MLIs showed SR-S in the PSTH peak (−16.4 ± 1.2%, *n* = 5, *p* = 0.03), but not in basal frequency (−1.2 ± 8.6%, *n* = 5, *p* = 0.8; Figures 4A,B). Thus, NMDA receptors appear to play a role in modifying TSS-induced PSTH-peaks as well as basal frequency of PC units and generating variability in basal frequency of MLIs, suggesting a differential control over basal and stimulus-evoked discharges.

Since long-term synaptic plasticity in the cerebellar cortex may also involve the inhibitory circuits both in the granular and molecular layer (Jörntell and Ekerot, 2002; Roggeri et al., 2008), we next tested whether blocking GABA-A receptors by applying gabazine (100 μM) on the cerebellar surface affected SR-P and/or SR-S induced by TSS (Figure 4A). During these experiments we identified eight PC units and eight MLI units. In the presence of gabazine, all the PC and MLI units showed a general increase in PSTH peak (PC 30.9 ± 4.9%; *n* = 8, *p* = 0.0004; MLI 35.2 ± 5.8%, *n* = 8, *p* = 0.002) and in basal frequency (PC 39.6.5 ± 6.5%; *n* = 8, *p* = 0.007; MLI 45.6 ± 8.8%, *n* = 8, *p* = 0.008) before TSS was applied. Following TSS in the presence of gabazine, all the PC units showed SR-P in the PSTH peak (Figures 4A,B; peak amplitude 49.4 ± 0.6%, *n* = 8, *p* = 0.003), but the changes in basal firing frequency were too variable to reach significance (−4.2 ± 5.3%; *n* = 8, *p* = 0.8). In MLI units, applying gabazine after TSS prevented SR-S in most of the units (peak amplitude −6.1 ± 6.9%, *n* = 8, *p* = 0.8; basal firing frequency 6.6 ± 4.7%, *n* = 8, *p* = 0.7).

In summary, in response to sensory stimulation, APV, but not gabazine, prevented SR-P in PC units, whereas gabazine, but not APV, prevented SR-S in MLI units, further differentiating PCs from MLIs based on their pharmacological plasticity profiles and highlighting the importance of these profiles in mediating synergy in the overall long-term plasticity during natural entrainment. The basal frequency changes were usually smaller (if significant) than those in PSTH peak amplitude changes, suggesting that regulation of the probability of discharge in response to sensory inputs and the basal neuronal frequency were at least in part diversified. The statistical comparison of all data (multiple ANOVA) confirmed the difference among the groups identified above (Figure 4C).

## Discussion

The main observation reported in this article is that naturally patterned sensory entrainment consistently induces long-term changes of spike discharge in PCs and the neurons that inhibit them, i.e., MLIs. All PCs showed an increase in SS responses (i.e., SR-P) over more than 30 min, whereas all MLIs showed a suppression in their responses (i.e., SR-S) over the same period. Further insight was provided by the different organization of plasticity observed following PF ES with TBS. In this case, just 56% of PCs showed SR-P, whereas the others showed SR-S. Moreover, consensual changes in basal firing frequency of PCs and MLIs went along with those in spike response probability only following tactile but not following ES. The different impact of sensory *vs.* electrical stimulation suggests that long-term modifications of PC discharge reflect a combination of mechanisms located not only at the PF-PC relay but also at other sites distributed across the granular and molecular layer (Hansel et al., 2001; Gao et al., 2012; D’Angelo, 2014). Eventually, all the putative changes should concur synergistically to induce robust SR-P in PCs at the end-point of the neuronal chain of the cerebellar cortex.

### Possible Plasticity Mechanisms Engaged *In Vivo* Along the Mossy Fiber-Parallel Fiber Pathway

While this work illustrates the global effect of theta stimulation patterns *in vivo*, the cellular processes that could have occurred along the MF pathway up to PCs cannot be directly dissected here but can be hypothesized by the analysis of experiments previously carried out in acute cerebellar slices. In response to punctuate facial stimulation, MFs are known to discharge in short spike bursts causing retransmission of new bursts from granule cells (Jaeger and Bower, 1994; Chadderton et al., 2004). These bursts, once organized in theta-patterns, have been shown *ex vivo* to engage a series of synaptic mechanisms including: (1) MF-GrC LTP (Roggeri et al., 2008); (2) presynaptic PF-PC LTP (Qiu and Knöpfel, 2007); (3) postsynaptic PF-PC LTP (Lev-Ram et al., 2003; Coesmans et al., 2004); and (4) PF-MLI LTD (Tanaka et al., 2013). Moreover, patterned synaptic stimulation was shown to cause changes in intrinsic excitability in granule cells (Armano et al., 2000) and PCs (Belmeguenai et al., 2010).

There are actually three experimental stand-points that help identifying the possible plasticity mechanisms occurring in the network. First, given the opposite sign of plasticity in PCs and MLIs, it is likely that SR-P in PCs following natural sensory training results, at least in part, from reduced MLI inhibition, akin with observations in decerebrated cats (Jörntell and Ekerot, 2002). Secondly, although a change in CF activation might have contributed to plasticity in the cerebellar cortex during the actual entrainment (Mathy et al., 2009; Gao et al., 2012), it is unlikely that it continued to do so after the entrainment, because CS frequency was not significantly altered in the post-TBS period. Thirdly, the granular layer could be important (if not critical) to shape PCs toward SR-P. Actually, changes both in synaptic transmission and intrinsic excitability occur in the granular layer following patterned TS (Roggeri et al., 2008) and their coexistence has been demonstrated trough mathematical reconvolution of LFPs *in vivo* (Diwakar et al., 2011). This hypothesis has been further investigated through pharmacological tests (see below). Therefore, although the potential role of changes in PC intrinsic excitability cannot be dismissed (Johansson et al., 2014), it appears to be just one in a more extended set of changes.

### NMDA and GABA-A Receptor Blockage Supports the Intervention of Granular Layer Plasticity

Pharmacological analysis was performed by superfusing the inhibitors of NMDA or GABA-A receptors, APV and gabazine, which were previously shown to diffuse to the granular layer where they effectively inhibit local receptor functions (Roggeri et al., 2008). The main effect of the NMDA receptor blocker APV on plasticity was to turn SR-P into SR-S in PC units. It seems unlikely that APV acted by blocking PF NMDA-receptor-dependent LTD (Casado et al., 2002), since this would prevent presynaptic induction of LTD and thereby enhance rather than suppress PC firing. Similarly, a block of NMDA receptors at PF-MLI synapses (Carter and Regehr, 2000) would reduce MLI activation and PC inhibition thereby enhancing rather than suppressing PC firing. Then the primary action of APV might have been on granule cells through a blockage of postsynaptic NMDA receptors at mossy fiber synapses (D’Errico et al., 2009) in the granular layer (Roggeri et al., 2008) and through a blockage of presynaptic NMDA receptor-dependent LTP at PF synapses in the molecular layer (Qiu and Knöpfel, 2007).

The main effect on plasticity of the GABA-A receptor blocker gabazine was to prevent SR-S in MLI units. One possibility is that LTP at MF to granule cell synapses following TSS (Roggeri et al., 2008) overexcites MLIs masking SR-S. Another possibility is that MLIs are overexcited due to the loss of GABAergic self-inhibition in the MLI network (Wulff et al., 2007; Rossi et al., 2012), again resulting in a reduced SR-S in MLIs. Thus, the prevalent occurrence of PC SR-P normally observed following TSS probably reflects, at least in part, plastic changes in the granular layer and is in line with the hypothesis that a suppression of MLI activity contributes to PC SR-P, akin with previous observations in decerebrated cats (Jörntell and Ekerot, 2002).

The question then arises as to how patterned activity in the mossy fiber pathway might coordinate different forms of plasticity at different sites in the granular and molecular layer. A global coordinating action could be exerted by NO, a non-conventional gaseous transmitter, which is released in an NMDA receptor-dependent manner both in the granular and molecular layer where it can strongly influence plasticity (Maffei et al., 2003; D’Angelo, 2014). A second set of factors may reside in the highly organized interplay between NMDA and GABA receptor systems (Bidoret et al., 2009; Rossi et al., 2012), which were shown to regulate the PC output in a time- and frequency-dependent manner (D’Angelo and De Zeeuw, 2009; Mapelli et al., 2010) and turn out now to coordinate the sign of long-term changes in PC spike-related discharge. Therefore, the intimate relationship between principal neurons and interneurons that was suggested to regulate neuronal synaptic responsiveness in the cerebral cortex (Marder and Buonomano, 2004; Klyachko and Stevens, 2006) may also be critical in the cerebellum (Jörntell and Ekerot, 2002).

### Theoretical Implications

The convergence of circuit modifications toward a potentiation of PC spike discharge fits with the theoretical expectation of PF-PC LTP when a mossy fiber input occurs without climbing fiber supervision (Sakurai, 1987; Lev-Ram et al., 2002), while PF-PC LTD should be driven by error–related signals carried by CFs (Ito and Kano, 1982). Our data suggest, however, that it would be more appropriate to consider that multiple mechanisms contribute to determine the sign of PC spike discharge changes rather than referring only to the PF-PC synapse. In this respect, it may also be relevant to consider a role for the projection from the cerebellar nuclei into the granular layer (Houck and Person, 2014; Ankri et al., 2015; Gao et al., 2016), which may serve as a feedback of PC processing, mediating consolidation effects. In a behavioral context, such a coordinated plasticity mechanism could further stabilize the effect of experience-dependent firing patterns in the cerebellum. It remains to be determined whether the schemes for memory formation in the cerebellar circuit coincide or diverge from those identified for more intensely investigated regions like the hippocampus and cerebral cortex (Lever et al., 2002; Dragoi et al., 2003; Hafting et al., 2008).

## Conclusion

In conclusion, sensory stimulation can induce coordinated plastic changes in the cerebellar cortical network *in vivo* in the absence of the intervention of CFs. These changes appear to involve several synapses and neurons located both in the granular and molecular layer, eventually determining synergistic modifications potentiating PC basal discharge as well as PC spike generation in response to mossy fiber inputs. As a consequence, the fine-tuning of PC spike emission on the millisecond time-scale could provide an efficient mechanism for regulating the cerebellar output and the behavioral response. While the intervention of PC and MLI synapses clearly emerged from this study, the further identification of different components of plasticity would require extensive experimental work *in slices* and *in vivo* using a combination of electrophysiological and optogenetics techniques as well as modeling of the spatio-temporal dynamics of multi-site plasticity in the network.

## Author Contributions

KBR performed the experimental work and analyzed the data. KV and LDP performed advanced data analysis. ED’A and CIDZ coordinated the work and provided the research infrastructures.

## Conflict of Interest Statement

The authors declare that the research was conducted in the absence of any commercial or financial relationships that could be construed as a potential conflict of interest. The current study used Spike-Train, which is software for spike analyses developed by Neurasmus BV, a non-profit company of Erasmus MC.
